# Supplementary material for: Correction for both common and rare cell types in blood is important to identify genes that correlate with age
Source: BMC Genomics. 2021 Mar 15;22:184. doi: 10.1186/s12864-020-07344-w (PMC7958454; doi:10.1186/s12864-020-07344-w)
Supplement: Supplementary file 11 — Additional file 11: Figure S1A. Heatmap of extended model predictors. A heatmap of Spearman correlations between all cell type predictors used in the extended model (EM). Figure S1B. Correlations of selected variable counts with age. The Spearman correlations of selected variables - including measured or imputed cell counts - with age are presented, colored per cohort (see legend). See Results section for details. Figure S2. Correlation of Z-scores associated with IM and EM genes. A Pearson correlation of the Z-scores associated with both significant and not significant IM and EM genes is shown. The 45° diagonal is presented as dashed, the correlation line is in red. See Results section for details. Figure S3. Cohort-related, gene-specific ρ values. A) QQ plots used to evaluate the distribution pattern of cohort-related, gene-specific ρ values. B) Gene expression residuals decrease with the EM. Homoscedasticity was evaluated by correlating gene expression residuals from every model with age, and the absolute Spearman ⍴ values obtained after meta-analysis are reported for all genes minus the shared genes significantly associated with aging. See Figure 2 in the main text and Methods for details. Statistical significance was assessed with a paired, one-tailed Wilcoxon test. The stars indicate statistical significance: *** P ≤ 0.001, ** P ≤ 0.01, * P ≤ 0.05. LL, LifeLines DEEP; LLS, Leiden Longevity Study; NTR, Netherlands Twin Registry; RS, Rotterdam Study; EM, extended model; IM, initial model; IM no age, IM without age as covariate; EM no age, EM without age as covariate. Figure S4. scRNA-seq data-derived t-SNE plots reveal that IM-related aging genes are more likely cell type-specific marker genes. Mean expression levels of cell type marker genes among aging-related genes identified in the Initial Model (IM, left) and in the Extended Model (EM, right) are plotted. Where applicable, IM- and EM-related intensities for same cell types plots were compared through a Wilc [file 12864_2020_7344_MOESM11_ESM.docx]

**Figure S1A. Heatmap of extended model predictors.** A heatmap of Spearman correlations between all cell type predictors used in the extended model (EM).
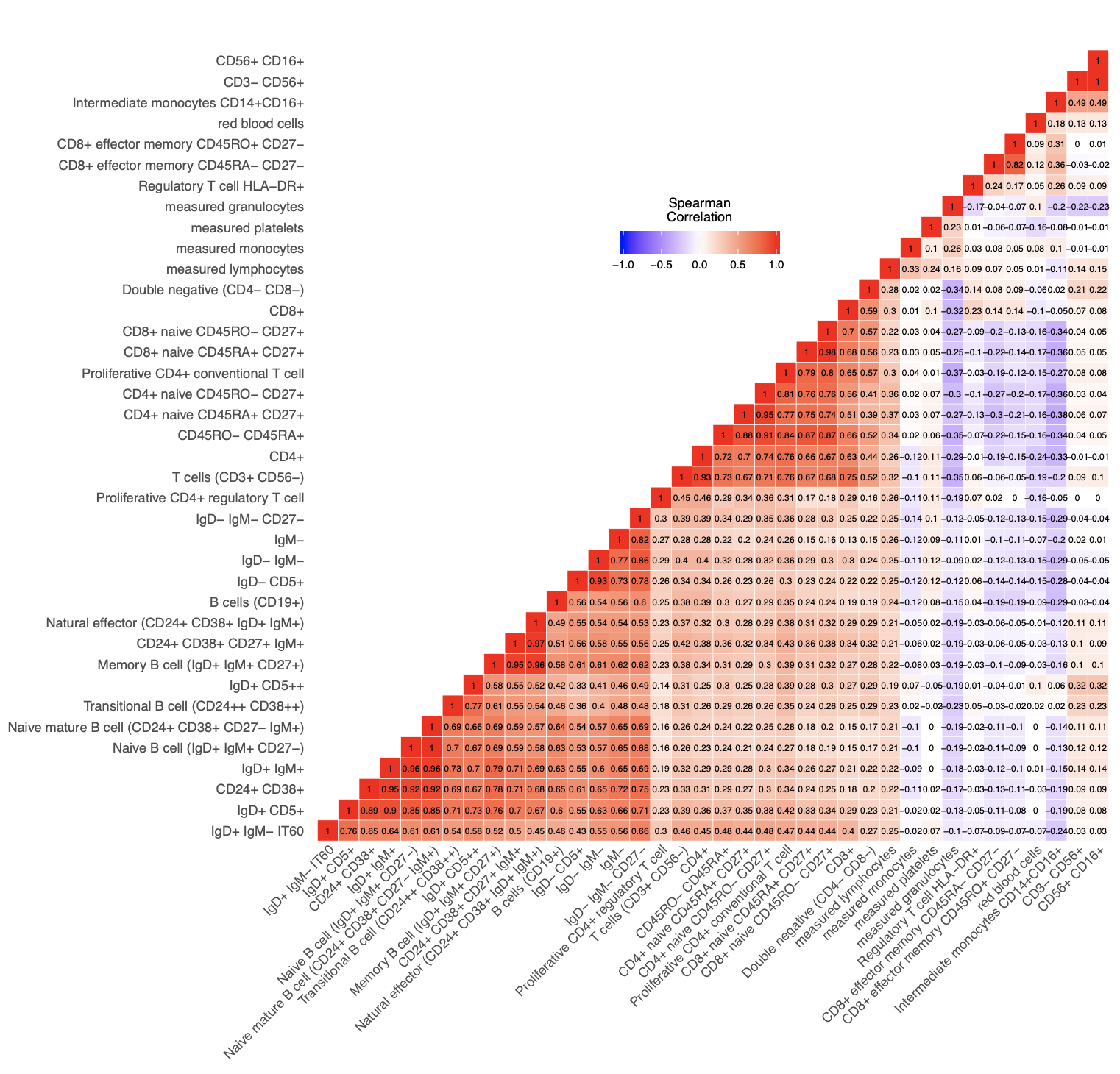


**
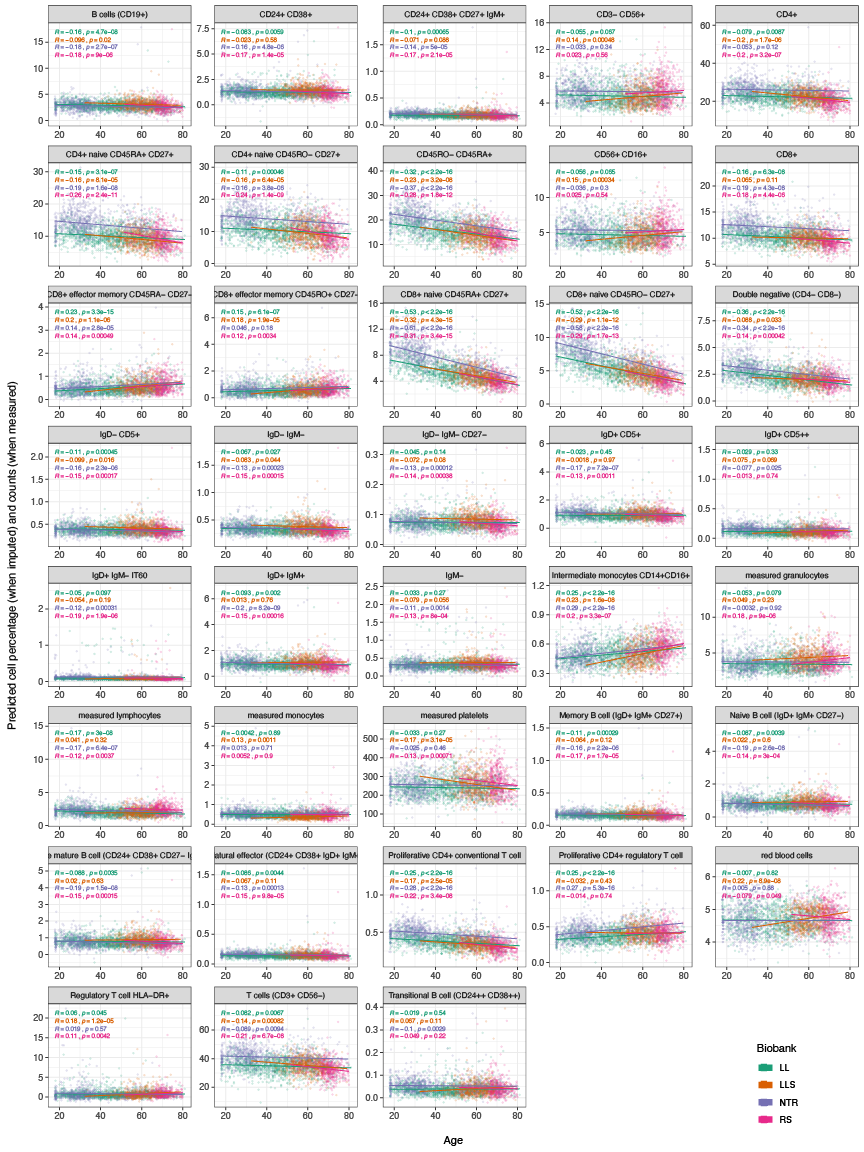
**

**Figure S1B. Correlations of selected variable counts with age.** The Spearman correlations of selected variables - including measured or imputed cell counts - with age are presented, colored per cohort (see legend). See *Results* section for details.


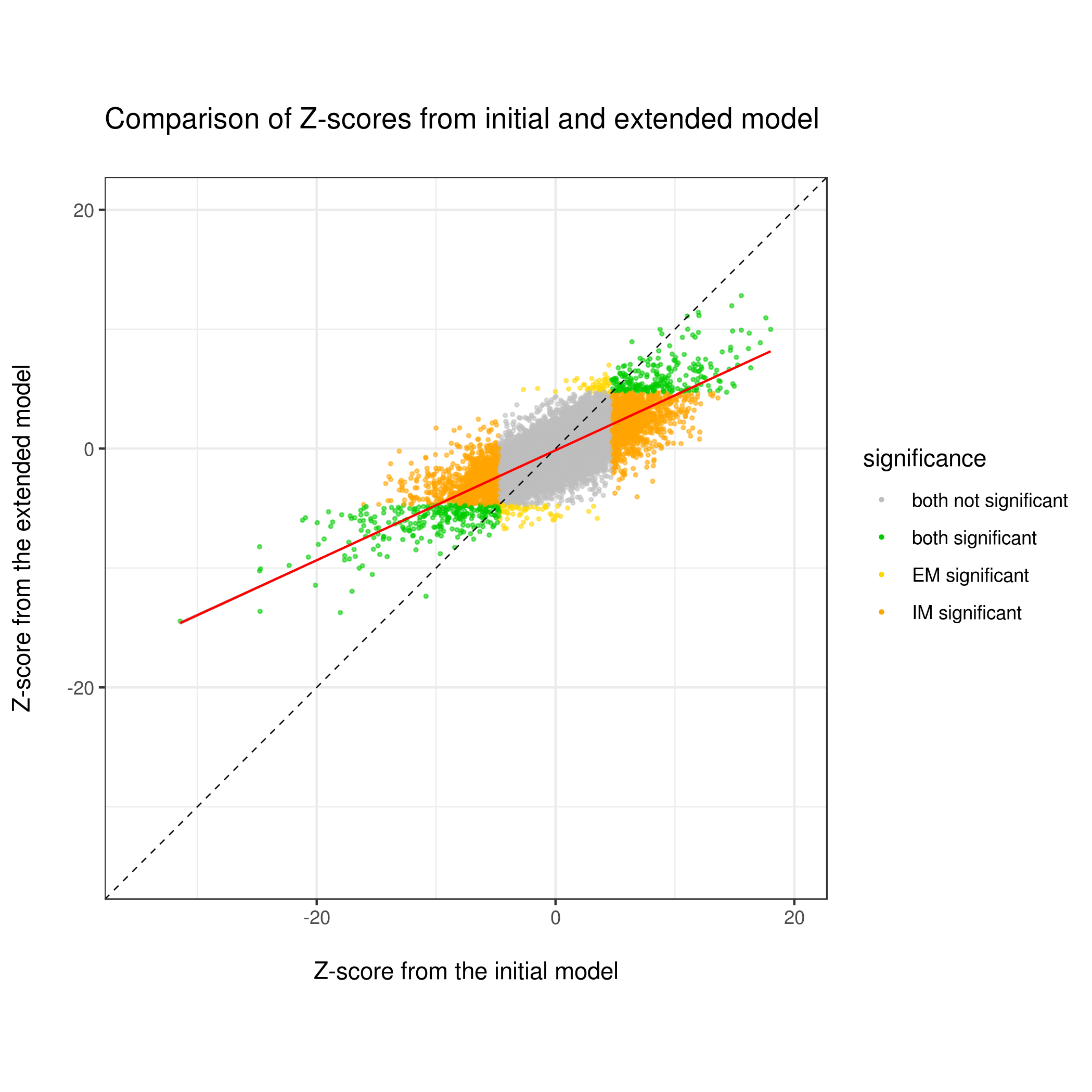


**Figure S2. Correlation of Z-scores associated with IM and EM genes.** A Pearson correlation of the Z-scores associated with both significant and not significant IM and EM genes is shown. The 45° diagonal is presented as dashed, the correlation line is in red. See *Results* section for details.

**Figure S3. Cohort-related, gene-specific ρ values.** A) QQ plots used to evaluate the distribution pattern of cohort-related, gene-specific ρ values. *
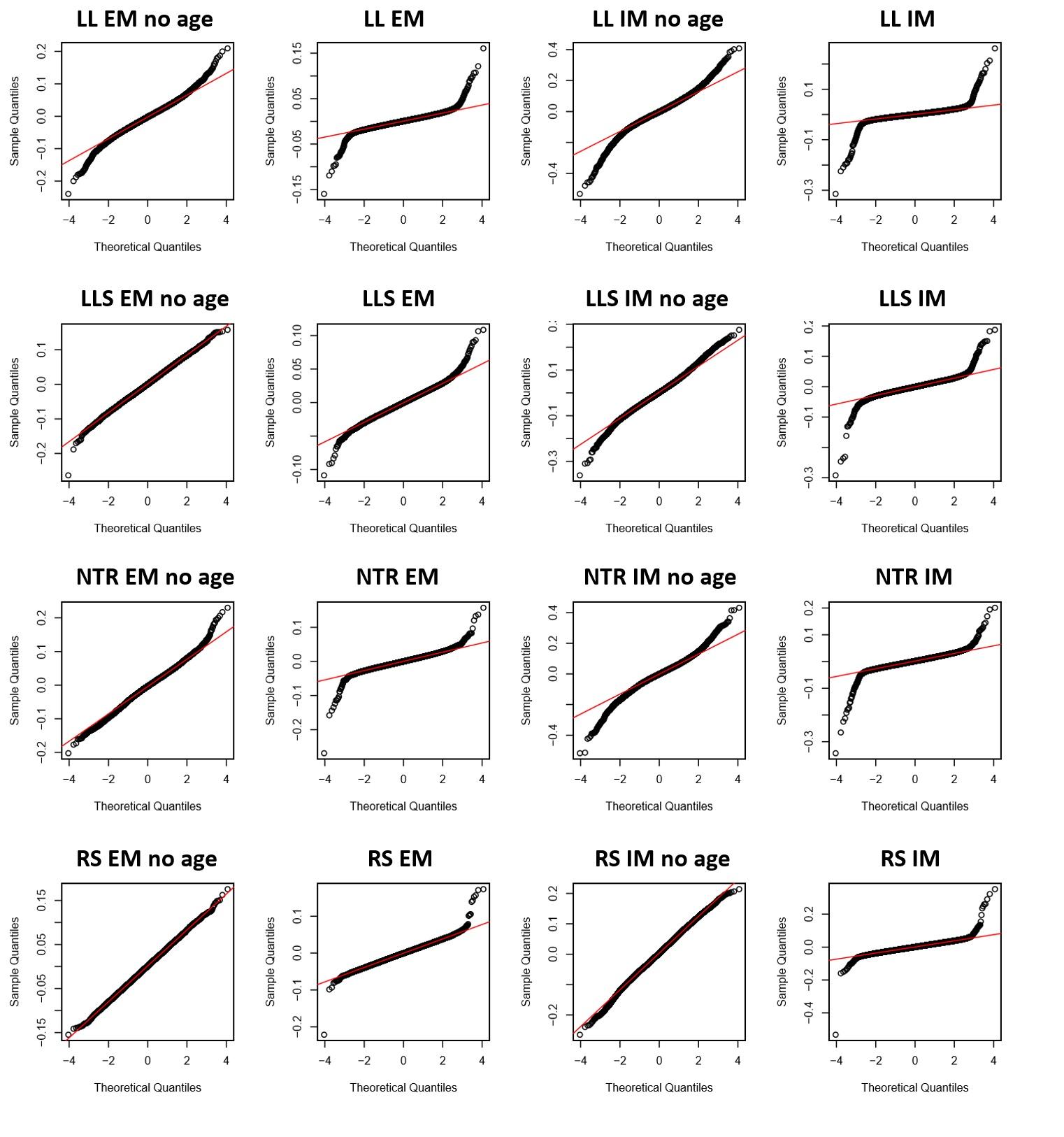
Continues*


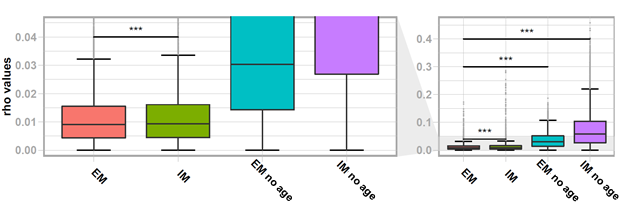


B) Gene expression residuals decrease with the EM. Homoscedasticity was evaluated by correlating gene expression residuals from every model with age, and the absolute Spearman ⍴ values obtained after meta-analysis are reported for all genes minus the shared genes significantly associated with aging. See Figure 2 in the main text and *Methods* for details. Statistical significance was assessed with a paired, one-tailed Wilcoxon test. The stars indicate statistical significance: *** P ≤ 0.001, ** P ≤ 0.01, * P ≤ 0.05. LL, LifeLines DEEP; LLS, Leiden Longevity Study; NTR, Netherlands Twin Registry; RS, Rotterdam Study; EM, extended model; IM, initial model; IM no age, IM without age as covariate; EM no age, EM without age as covariate. .

**Figure S4.** *Continues*
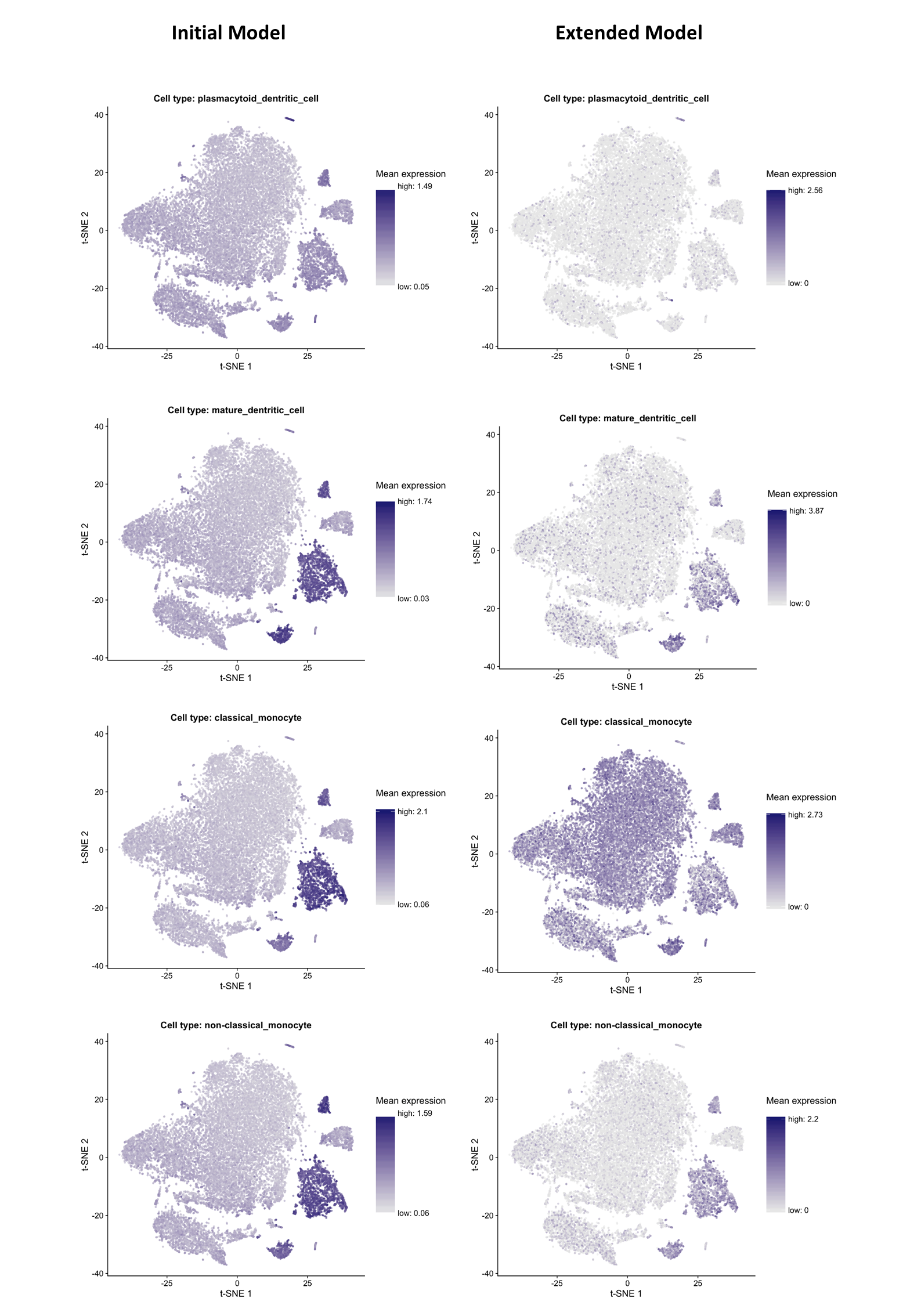


**Figure S4.** *Continues*
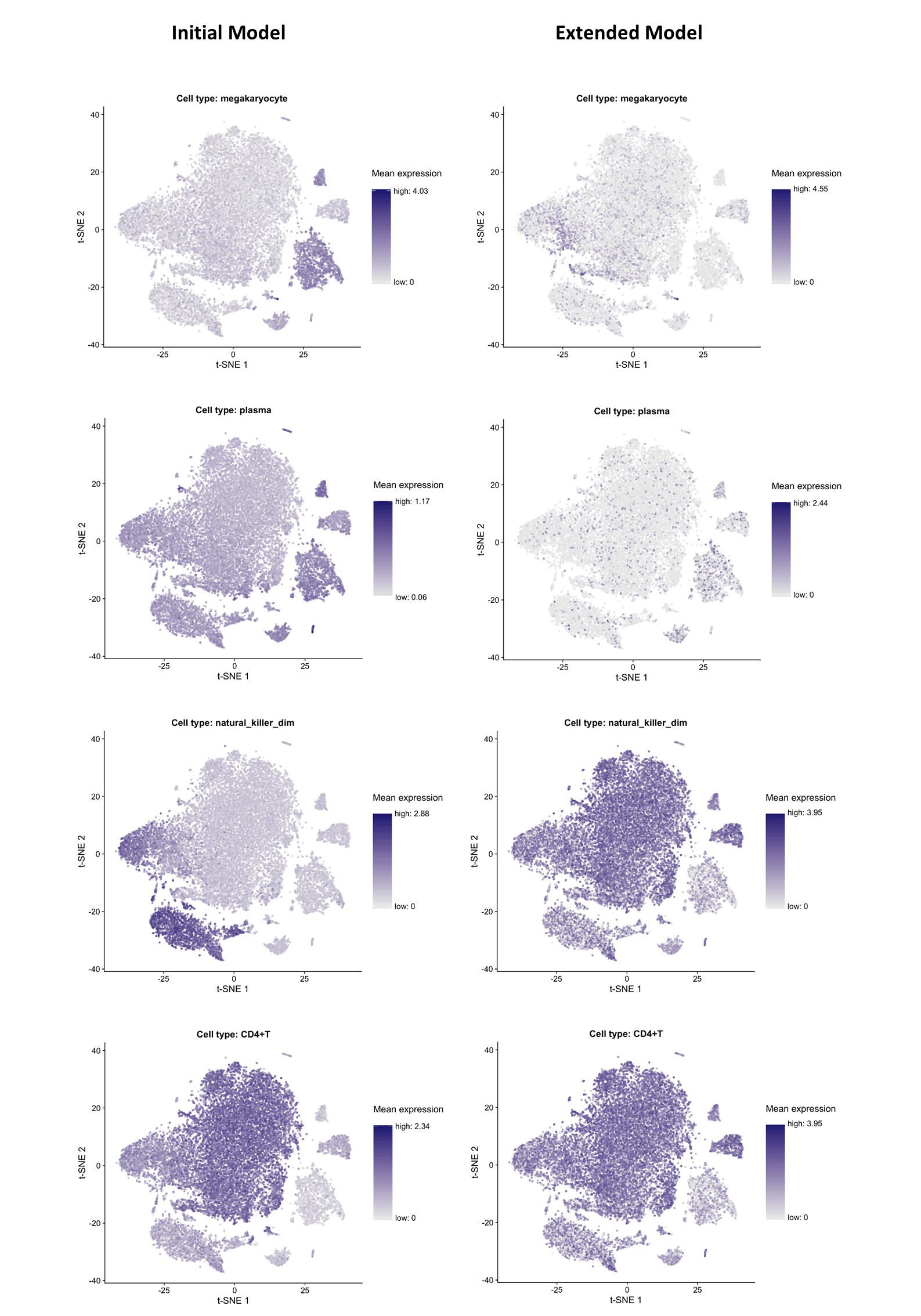


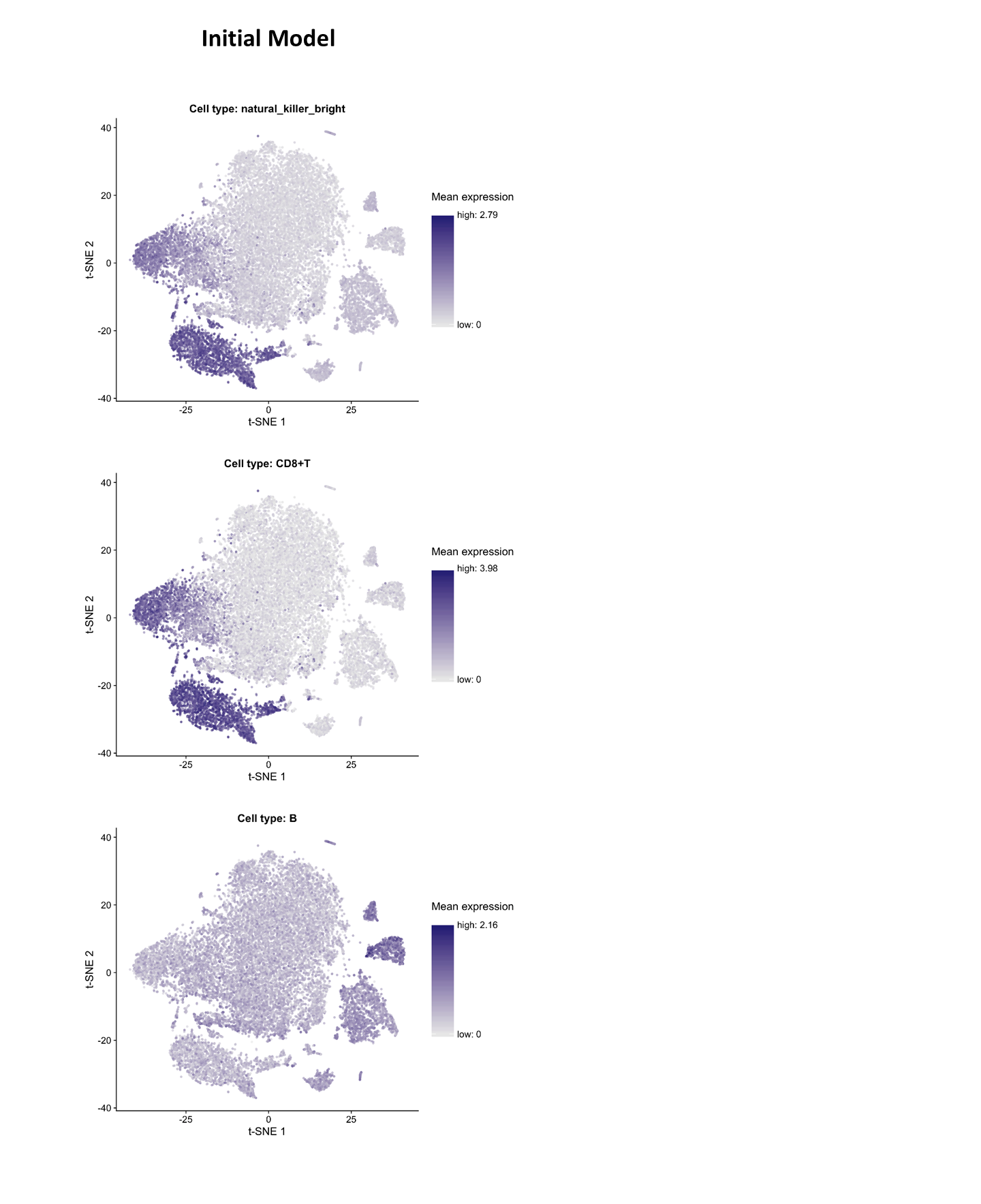
**Figure S4. scRNA-seq data-derived t-SNE plots reveal that IM-related aging genes are more likely cell type-specific marker genes.** Mean expression levels of cell type marker genes among aging-related genes identified in the Initial Model (IM, left) and in the Extended Model (EM, right) are plotted. Where applicable, IM- and EM-related intensities for same cell types plots were compared through a Wilcoxon test, always observing a P ≤ 2.2⨯10^-16^. For details regarding cell population-specific regions, refer to [20].


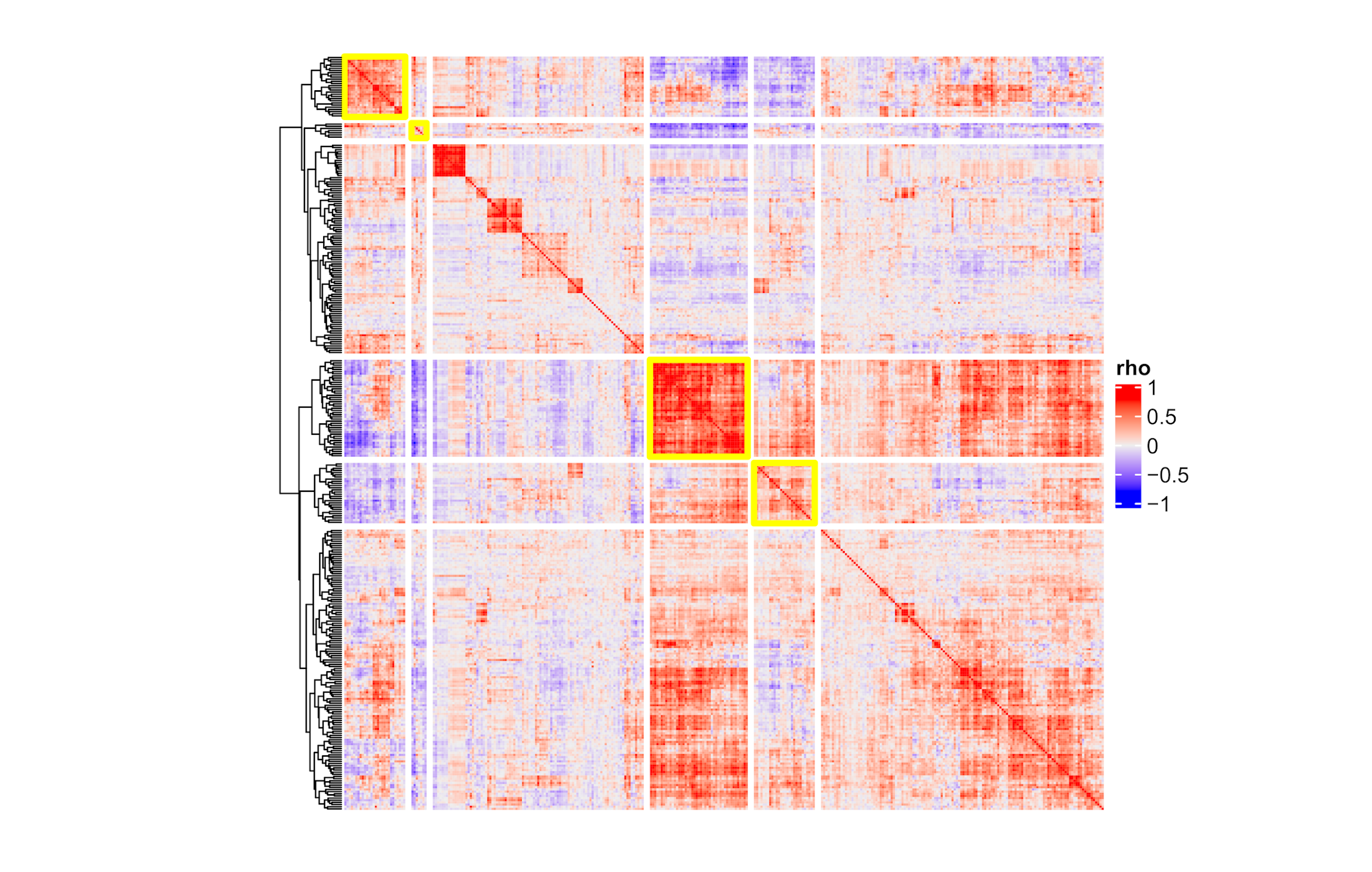


**Figure S5. Heatmap of gene expression residuals correlations for EM downregulated aging-related genes.** Heatmap showing downregulated EM aging-related genes clustered based on the paired correlation values of their gene expression residuals. Highly correlated clusters were identified and highlighted with a yellow border. See *Results* and *Methods* sections for details.


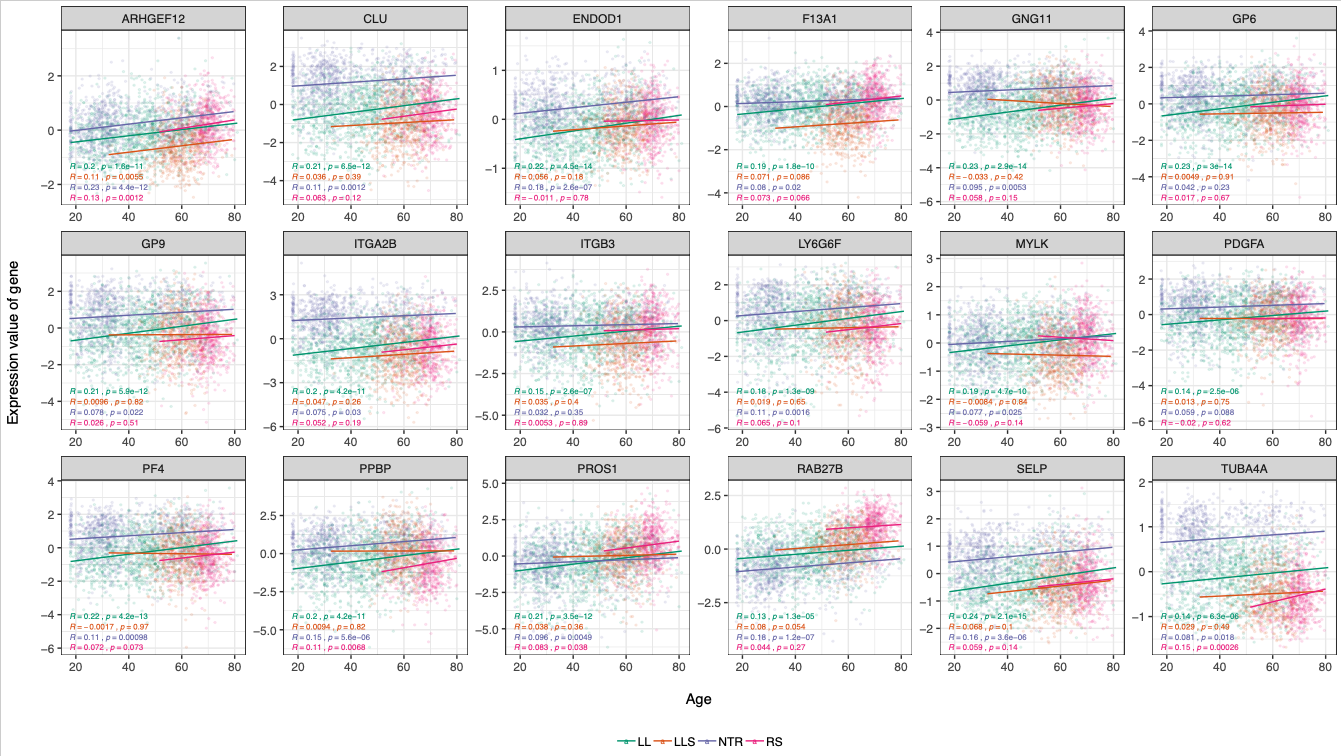


**Figure S6. Expression values of platelet-related genes across age.** The four cohorts are colored separately and the Spearman correlations and P-values are calculated for each cohort independently.


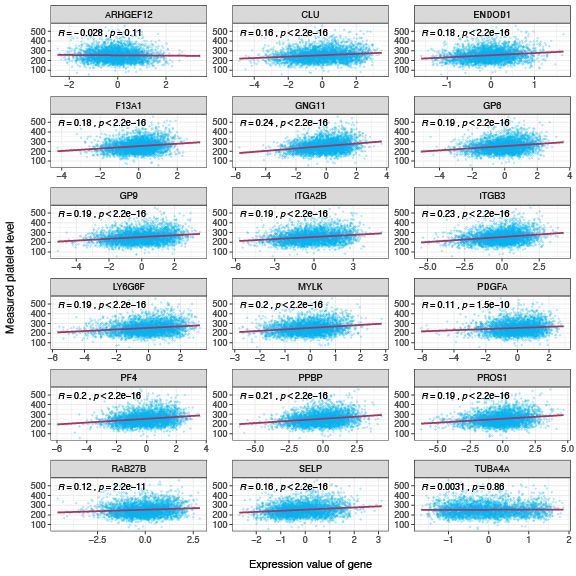


**Figure S7.** *Continues*


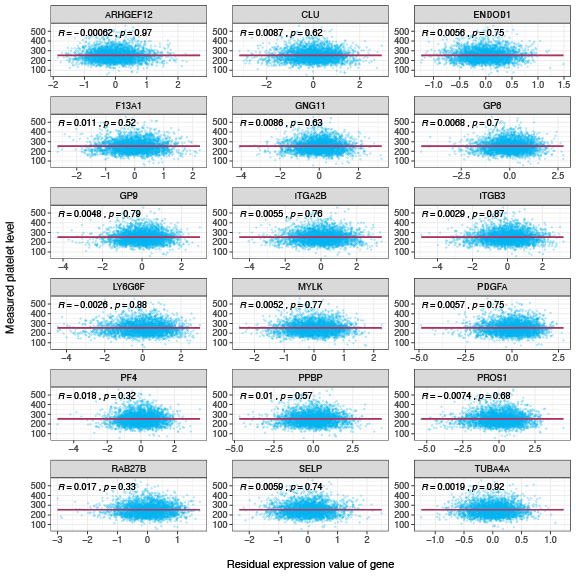


**Figure S7. Correlations between (residual) gene expression levels of the genes from platelet-related cluster 1 and measured platelet levels.** A) Spearman correlations between gene expression levels and measured platelets. B) Spearman correlations between gene expression residuals from the extended model and measured platelets.


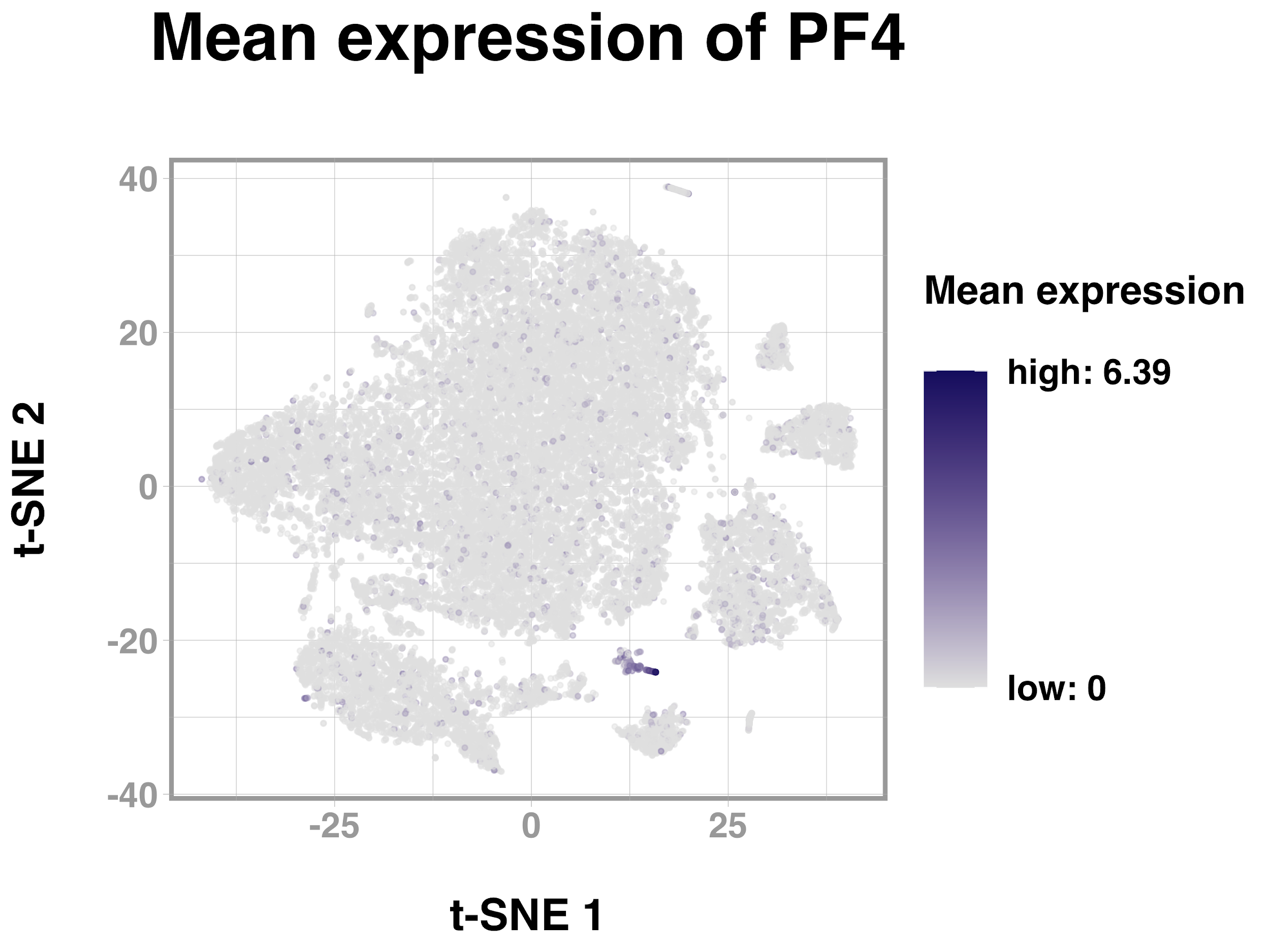


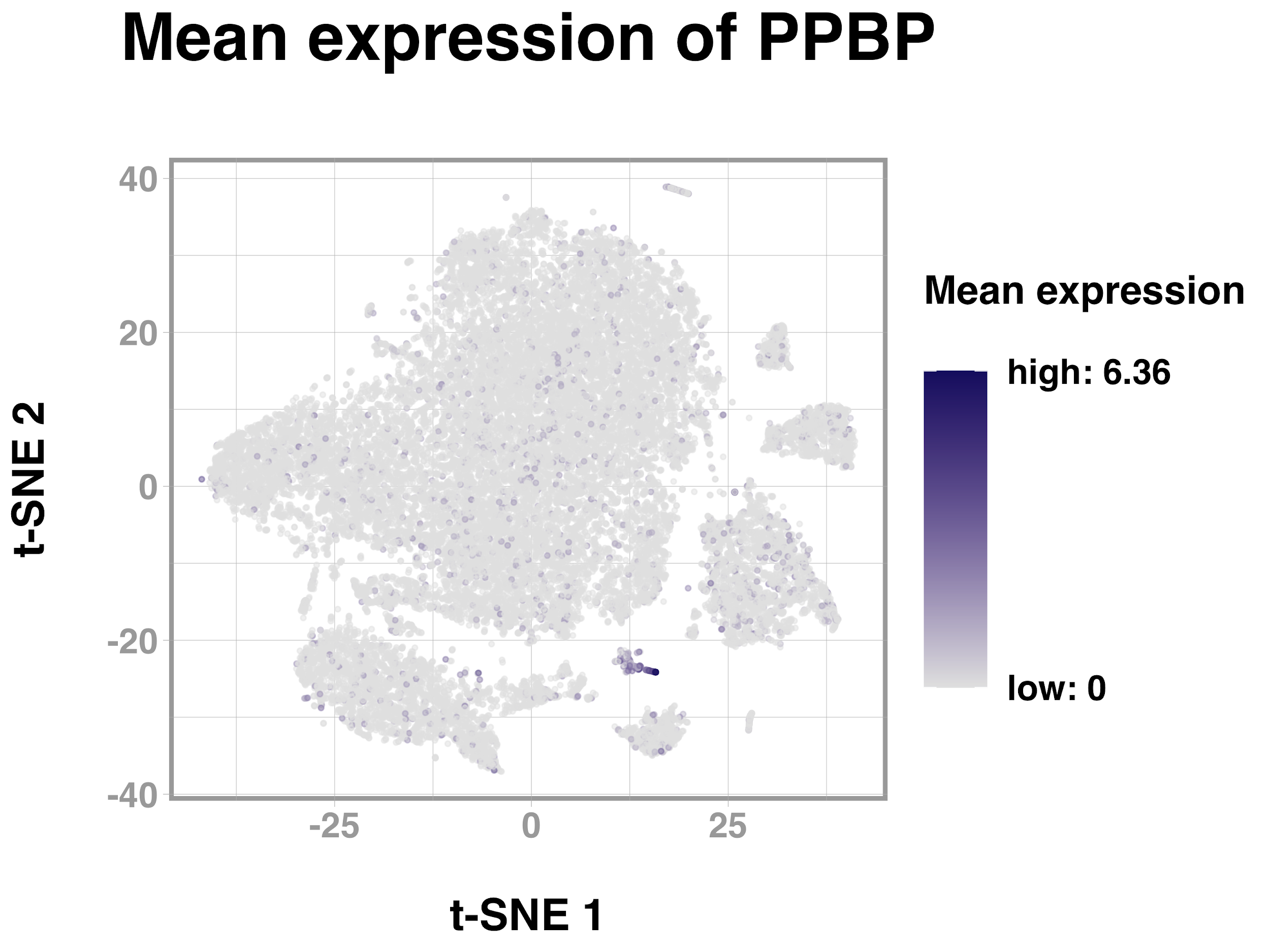


**Figure S8. scRNA-seq data-derived t-SNE plots reveal that *PF4* and *PPBP* are specifically expressed in megakaryocytes.** Mean expression levels of platelet marker genes *PF4* and *PPBP* are plotted. For details regarding cell population-specific regions, refer to [20].
